# Integrated biomarker model based on cTnI, BNP, and D-dimer for early risk stratification in acute aortic dissection

**DOI:** 10.3389/fcvm.2026.1926181

**Published:** 2026-08-27

**Authors:** Yanfen Yao, Guochen Li, Li Kong

**Affiliations:** 1 The First Clinical Medical College, Shandong University of Traditional Chinese Medicine, Jinan, Shandong, China; 2 Department of Intensive Care Medicine, Shandong Provincial Third Hospital, Shandong University, Jinan, Shandong, China; 3 Department of Emergency Intensive Care Medical Center, Shandong University of Traditional Chinese Medicine Affiliated Hospital, Jinan, Shandong, China; 4 Department of Emergency Center, Shandong University of Traditional Chinese Medicine Affiliated Hospital, Jinan, Shandong, China

**Keywords:** aortic dissection, prognostic model, biomarkers, cTnI, BNP, D-dimer, 30-day mortality

## Abstract

**Background:**

Early identification of patients at high risk of death following acute aortic dissection (AAD) remains a major clinical challenge because mortality is driven by multiple interacting pathophysiological mechanisms, including myocardial injury, hemodynamic stress, and activation of coagulation pathways. We aimed to develop and internally validate an integrated biomarker-based model for predicting 30-day mortality in patients with AAD.

**Methods:**

We conducted a retrospective cohort study to develop and internally validate a multivariable prediction model for 30-day mortality in 572 consecutive patients with AAD. Admission levels of cardiac troponin I (cTnI), creatine kinase-MB (CK-MB), B-type natriuretic peptide (BNP), D-dimer, and fibrin/fibrinogen degradation products (FDP) were assessed. Multivariable logistic regression was performed after adjustment for age, Stanford classification, symptom-onset-to-admission time, systolic blood pressure, serum creatinine, and treatment strategy. Model performance was evaluated using discrimination, calibration, net reclassification improvement (NRI), and integrated discrimination improvement (IDI). Internal validation was conducted using bootstrap resampling with 1,000 iterations.

**Results:**

Elevated cTnI (adjusted OR = 3.76, 95% CI: 1.87–7.56; *P* < 0.001), BNP (adjusted OR = 6.59, 95% CI: 2.76–15.76; *P* < 0.001), and D-dimer (adjusted OR = 2.70, 95% CI: 1.17–6.24; *P* = 0.020) were independently associated with 30-day mortality, whereas CK-MB and FDP were not independently associated with mortality after multivariable adjustment. The integrated prediction model incorporating cTnI, BNP, D-dimer, and clinical covariates demonstrated good discrimination, with an optimism-corrected AUC of 0.89 compared with 0.84 for the clinical model alone, representing a modest but statistically significant improvement (ΔAUC = 0.05). At the optimal probability threshold, the integrated model achieved a sensitivity of 76.2% and a specificity of 96.8%. It also significantly improved risk classification over the clinical model alone, with an NRI of 0.32 (*P* < 0.01) and an IDI of 0.11 (*P* < 0.01). A nomogram derived from the final multivariable model was developed to facilitate individualized estimation of 30-day mortality risk using routinely available clinical and laboratory variables.

**Conclusions:**

An integrated model incorporating cTnI, BNP, D-dimer, and key clinical variables demonstrated good discrimination (optimism-corrected AUC 0.89) and statistically significant incremental value over clinical variables alone (NRI = 0.32, IDI = 0.11). However, the modest AUC improvement (ΔAUC = 0.05) and lack of head-to-head comparison with established risk scores (IRAD, Penn, GERAADA) warrant cautious interpretation and external validation before clinical implementation.

## Introduction

Acute aortic dissection (AAD) is a catastrophic cardiovascular emergency characterized by disruption of the aortic intima, permitting blood to enter the medial layer and create a false lumen (1, 2). Despite advances in diagnostic imaging, perioperative management, and surgical techniques, AAD continues to be associated with substantial morbidity and mortality (3). Mortality increases rapidly during the early phase of the disease, particularly among patients with Stanford type A dissection, emphasizing the importance of prompt diagnosis and risk assessment (4). Even among patients receiving contemporary treatment, adverse outcomes remain common because of complications such as aortic rupture, cardiac tamponade, malperfusion syndromes, myocardial ischemia, and multi organ failure (5).

Accurate early risk stratification is essential for identifying patients at increased risk of death and guiding decisions regarding surgical intervention, intensive monitoring, and allocation of healthcare resources (6). Current prognostic assessment in AAD relies primarily on demographic characteristics, hemodynamic status, imaging findings, and clinical presentation (7, 8). Established predictors of adverse outcomes include advanced age, hypotension, organ malperfusion, renal dysfunction, and dissection extent (9). However, clinical variables alone may not fully capture the complex biological processes underlying disease progression. Consequently, there has been growing interest in the use of circulating biomarkers as objective tools for improving prognostic assessment in patients with AAD.

Several pathophysiological mechanisms contribute to adverse outcomes following AAD, including myocardial injury, hemodynamic stress, inflammation, and activation of coagulation and fibrinolytic pathways (10). Cardiac troponin I (cTnI) is a highly sensitive marker of myocardial injury and may become elevated in patients with coronary malperfusion, myocardial ischemia, or severe hemodynamic compromise (11). B-type natriuretic peptide (BNP) reflects ventricular wall stress and neurohormonal activation and may increase in the presence of acute aortic regurgitation, heart failure, or cardiac tamponade (12). D-dimer, a fibrin degradation product generated during fibrinolysis, reflects activation of coagulation pathways and has been extensively investigated as both a diagnostic and prognostic biomarker in AAD (13). Previous studies have demonstrated associations between elevated concentrations of these biomarkers and increased mortality risk; however, their prognostic utility has predominantly been evaluated individually. Importantly, the incremental prognostic value of combining these biomarkers with established clinical predictors remains incompletely characterized, and concerns regarding model overfitting have not been systematically addressed (14).

Because these biomarkers represent distinct but complementary biological pathways, a multimarker approach may provide more comprehensive prognostic information than any single biomarker alone. Multi biomarker strategies have improved risk prediction in several cardiovascular conditions, including acute coronary syndromes and heart failure, yet evidence supporting their use in AAD remains limited (15). Furthermore, the incremental prognostic value of combining biomarkers with established clinical predictors has not been fully elucidated. Additional biomarkers associated with myocardial injury and coagulation activation, including creatine kinase-MB (CK-MB) and fibrin/fibrinogen degradation products (FDP), have also been investigated in cardiovascular disease, but their independent contribution to risk prediction in AAD remains uncertain.

Accordingly, the present study aimed to evaluate the prognostic significance of admission cTnI, BNP, D-dimer, CK-MB, and FDP levels in patients with acute aortic dissection and to develop an integrated prediction model for 30-day all-cause mortality. We further sought to determine whether a multimarker strategy provides incremental prognostic value beyond conventional clinical variables and to construct a clinically applicable risk prediction tool for bedside assessment. We hypothesized that combining biomarkers reflecting myocardial injury, hemodynamic stress, and coagulation activation would improve early risk stratification compared with clinical assessment alone.

## Methods

### Study design and setting

This retrospective observational cohort study was conducted at a tertiary academic referral center between January 2020 and December 2024. The study was designed to evaluate the prognostic value of admission biomarkers and to develop an integrated prediction model for 30-day all-cause mortality in patients with AAD. The study was conducted and reported in accordance with the Strengthening the Reporting of Observational Studies in Epidemiology (STROBE) statement and the Transparent Reporting of a Multivariable Prediction Model for Individual Prognosis or Diagnosis (TRIPOD) recommendations.

### Study population

Consecutive adult patients diagnosed with AAD during the study period were screened for eligibility. The diagnosis of AAD was confirmed by computed tomography angiography (CTA) and classified according to the Stanford classification system.

Eligible patients fulfilled all of the following criteria: (1) age ≥18 years; (2) confirmed diagnosis of Stanford type A or type B AAD; (3) symptom onset within 14 days before hospital admission; (4) availability of admission biomarker measurements, including cTnI, B-type natriuretic peptide (BNP), and D-dimer; and (5) availability of follow-up data for determination of 30-day mortality status.

**Figure 1:**
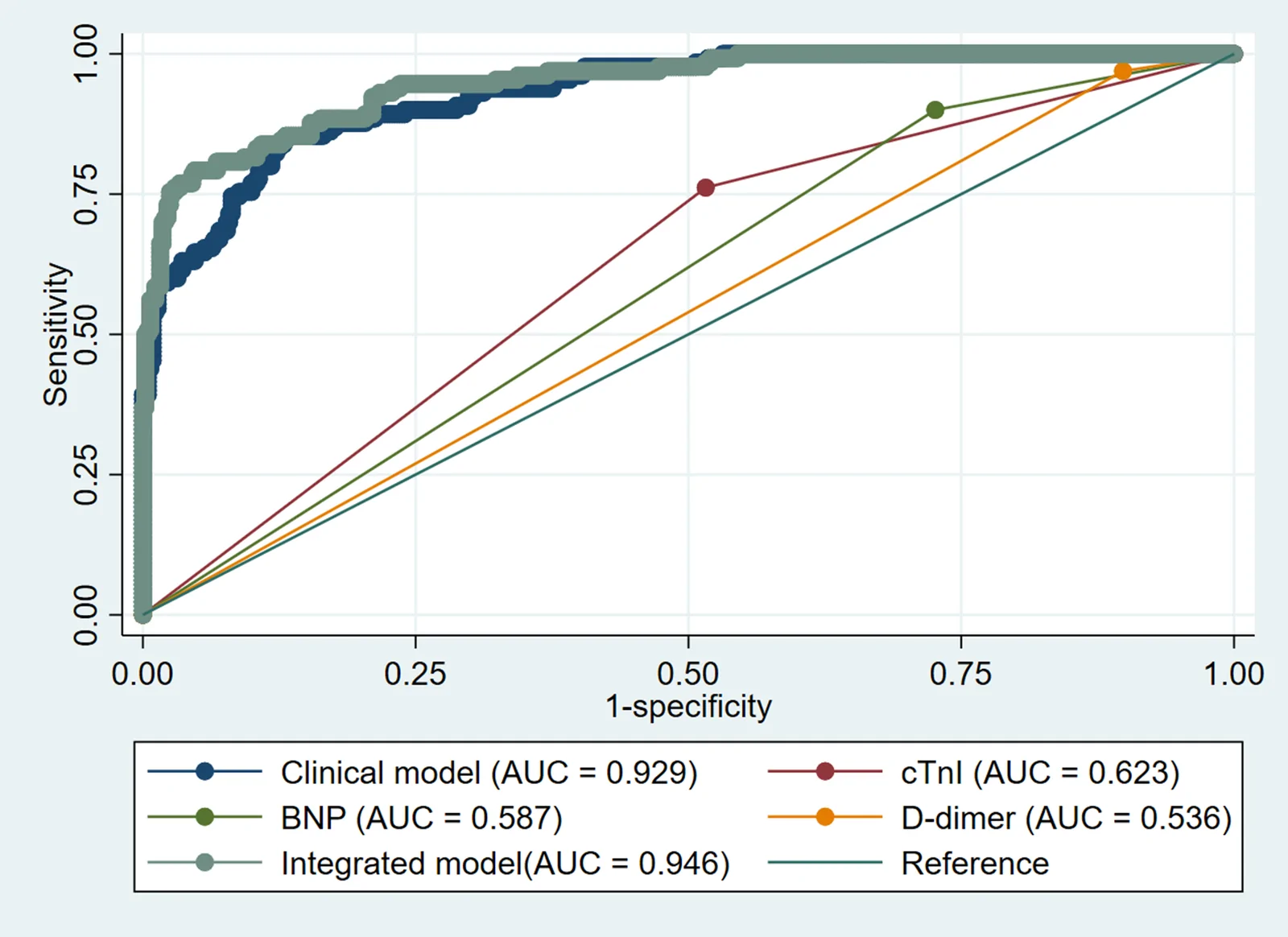
Study flow diagram.

Patients were excluded if they had: (1) chronic aortic dissection (>14 days from symptom onset); (2) traumatic or iatrogenic aortic dissection; (3) previous aortic surgery or endovascular aortic intervention; (4) pregnancy; (5) active malignancy with an expected survival of less than six months; (6) end-stage renal disease requiring chronic dialysis; or (7) incomplete data regarding the primary outcome or key study biomarkers. A total of 572 eligible patients were included in the final analysis (Figure 1, flowchart).

### Sample size considerations

As this was a retrospective observational study, no formal *a priori* sample size calculation was performed. The final cohort comprised 572 patients, including 130 deaths within 30 days of admission. Candidate predictors were selected based on clinical relevance and existing evidence regarding prognosis in AAD (16). The number of outcome events was considered sufficient for multivariable logistic regression modeling and development of the prognostic model. To further reduce the risk of model overfitting and assess model stability, internal validation was performed using bootstrap resampling. With 130 outcome events and 7 candidate predictors, the sample size exceeds the recommended minimum of 10 events per variable (EPV = 18.6), ensuring adequate power for model development.

### Data collection

Clinical data were extracted from the institutional electronic medical record system using a standardized data collection form. Baseline information was obtained at the time of hospital admission and included demographic characteristics, medical history, clinical presentation, physiological parameters, laboratory findings, imaging characteristics, treatment strategy, and clinical outcomes. Two investigators independently reviewed electronic medical records. Discrepancies were resolved by consensus with a third investigator.

Demographic variables included age, sex, body mass index, height, and weight. Medical history variables included hypertension, diabetes mellitus, dyslipidemia, smoking status, alcohol consumption, coronary artery disease, prior stroke, chronic kidney disease, chronic obstructive pulmonary disease, and Marfan syndrome.

Clinical presentation variables included symptom-onset-to-admission time, admission time, diagnostic delay, chest pain, back pain, syncope, dyspnea, neurological deficit, shock, and cardiac tamponade. Shock was defined as systolic blood pressure <90 mmHg at presentation.

Vital signs recorded at admission included systolic blood pressure, diastolic blood pressure, mean arterial pressure, heart rate, respiratory rate, body temperature, and oxygen saturation.

Imaging variables included Stanford classification, DeBakey classification, aortic rupture, malperfusion syndrome, pericardial effusion, pleural effusion, patent false lumen, and false lumen thrombosis.

Treatment strategy was categorized as surgical management, endovascular intervention, or medical therapy alone according to multidisciplinary team assessment and contemporary guideline recommendations.

### Outcome definition

The primary outcome was 30-day all-cause mortality, defined as death from any cause occurring within 30 days after hospital admission. Mortality status was determined from hospital records, follow-up documentation, and institutional mortality databases where available. Outcome data were available for all patients included in the final analysis.

### Biomarker assessment

Venous blood samples were collected at hospital admission and before definitive therapeutic intervention whenever clinically feasible. All laboratory analyses were performed in the institutional central laboratory according to standardized operating procedures and routine quality-control protocols. Biomarker measurements were obtained from the first available blood sample following presentation, thereby reflecting the patient’s initial physiological and hemodynamic status during the acute phase of the disease.

The primary biomarkers of interest were cTnI, BNP, and D-dimer. These biomarkers were selected *a priori* based on their biological plausibility and previously reported associations with adverse outcomes in acute aortic dissection and other acute cardiovascular conditions. Cardiac troponin I is a highly sensitive and specific marker of myocardial injury and may become elevated in patients with coronary malperfusion, myocardial ischemia, or severe hemodynamic compromise. B-type natriuretic peptide reflects ventricular wall stress and neurohormonal activation and has been associated with acute heart failure, aortic regurgitation, and adverse cardiovascular outcomes. D-dimer, a fibrin degradation product generated during fibrinolysis, reflects activation of coagulation and fibrinolytic pathways and has been extensively investigated as both a diagnostic and prognostic biomarker in patients with acute aortic syndromes.

Additional biomarkers evaluated included CK-MB and fibrin/fibrinogen degradation products (FDP). CK-MB was assessed as an alternative marker of myocardial injury, whereas FDP was included as an additional indicator of coagulation and fibrinolytic activation. These biomarkers were examined to determine whether they provided incremental prognostic information beyond the primary biomarker panel. Inflammatory markers (CRP, Procalcitonin, ESR, IL-6, TNF-α, MLR, NLR, PLR, SII) and other laboratory parameters were collected as part of routine clinical assessment and are reported for descriptive purposes only; they were not included in the primary prediction model. Additional cardiac and coagulation markers, including myoglobin, troponin, NT-proBNP, and LDH, were measured from the same admission blood samples using standardized laboratory methods. Troponin and NT-proBNP were measured using chemiluminescent immunoassays, myoglobin using immunoturbidimetric assay, and LDH using enzymatic rate methods. All assays were performed in the institutional central laboratory according to routine quality-control protocols.

For prognostic modeling and to facilitate clinical applicability, biomarker levels were analyzed as dichotomous variables using institutionally validated upper reference limits. Elevated biomarker concentrations were defined as cTnI > 0.04 ng/mL, CK-MB > 5.0 ng/mL, BNP > 100 pg/mL, D-dimer > 0.5 mg/L, and FDP > 5.0 μg/mL. These thresholds were selected according to manufacturer-recommended reference ranges, institutional laboratory standards, and values commonly adopted in previous cardiovascular biomarker studies. All biomarker classifications were prespecified before statistical analysis and applied uniformly across the study population.

### Covariates and predictor selection

Potential predictors were selected *a priori* based on published evidence, biological plausibility, and established prognostic relevance in AAD. Particular emphasis was placed on variables routinely available at the time of hospital presentation. Treatment strategy was included as a covariate in the full multivariable model to adjust for potential confounding. However, treatment was excluded from the final parsimonious nomogram because treatment decisions occur after initial risk stratification and are strongly influenced by clinical severity; including treatment in a pretreatment risk prediction tool would introduce confounding by indication and limit clinical applicability.

Clinical variables considered for model development included age, Stanford classification, symptom-onset-to-admission time, systolic blood pressure, serum creatinine concentration, and treatment strategy. Biomarker variables included cTnI, CK-MB, BNP, D-dimer, and FDP. Certain variables, including Stanford classification, were prespecified and retained in model development because of their established clinical relevance and recognized association with outcomes in acute aortic dissection, regardless of statistical significance in multivariable analyses.

These predictors were selected to represent key domains associated with adverse outcomes, including demographic risk, disease severity, organ dysfunction, treatment-related factors, myocardial injury, hemodynamic stress, and coagulation activation.

### Statistical analysis

All statistical analyses were performed using Stata/SE version 17.0 (StataCorp LLC, College Station, TX, USA). All tests were two-sided, and a *P* value <0.05 was considered statistically significant. Data completeness was assessed before statistical analysis. Missing data were minimal for all candidate variables: age (0%), sex (0%), Stanford classification (0%), systolic blood pressure (0.5%), creatinine (1.2%), cTnI (2.1%), BNP (2.8%), D-dimer (3.1%), CK-MB (3.1%), FDP (2.1%), and symptom-onset-to-admission time (0.9%). Because missingness was low (<5% for all variables), the primary analyses were based on complete-case data. As a sensitivity analysis, missing values were imputed using Multiple Imputation by Chained Equations with 20 imputed datasets. The estimated regression coefficients and model performance (including discrimination) were essentially unchanged compared with the complete-case analysis (Supplementary Table S2), indicating that missing data had a negligible impact on the study findings. Continuous variables were assessed for normality using the Shapiro–Wilk test and visual inspection of distribution plots. Normally distributed variables are presented as mean ± standard deviation (SD), whereas non-normally distributed variables are presented as median and interquartile range (IQR). Categorical variables are presented as frequencies and percentages. Comparisons between survivors and non-survivors were performed using Student’s *t*-test or the Mann–Whitney *U* test for continuous variables and the *χ*^2^ test or Fisher’s exact test for categorical variables, as appropriate.

Continuous variables were analyzed in their original scale for descriptive analyses. Biomarkers were subsequently categorized according to predefined clinical thresholds to facilitate model interpretation and clinical applicability.

Univariable logistic regression analyses were initially performed to evaluate associations between candidate predictors and 30-day mortality. Variables considered clinically relevant based on existing literature and expert knowledge were subsequently entered into a multivariable logistic regression model irrespective of univariable statistical significance. Associations are reported as odds ratios (ORs) with corresponding 95% confidence intervals (CIs).

Multicollinearity among candidate predictors was formally evaluated using two complementary approaches: (1) pairwise Spearman rank correlation coefficients, and (2) variance inflation factors (VIFs). Pairwise correlations between biomarkers ranged from *ρ* = 0.004 (CK-MB vs. FDP, *P* = 0.927) to *ρ* = 0.203 (BNP vs. D-dimer, *P* < 0.001). Notably, correlations between cTnI and CK-MB (*ρ* = 0.104, *P* = 0.013) and between D-dimer and FDP (*ρ* = 0.191, *P* < 0.001) were weak, indicating that collinearity does not explain the loss of independent prognostic significance for CK-MB and FDP. VIF values for all predictors ranged between 1.12 and 1.89, remaining well below the conservative threshold of 5. To further evaluate whether CK-MB and FDP contributed independent information, we performed stepwise model selection using the Akaike information criterion (AIC). Removing CK-MB and FDP did not significantly change the model’s AIC (ΔAIC = 1.8, *P* = 0.18) or optimism-corrected AUC (ΔAUC = 0.006, *P* = 0.42). These findings confirm that CK-MB and FDP provide little independent prognostic information beyond cTnI and D-dimer, respectively.

### Model development and performance assessment

A multivariable prediction model was developed using admission clinical variables and biomarker measurements. Model discrimination was assessed using the area under the receiver operating characteristic curve (AUC) with corresponding 95% confidence intervals. Differences in AUC between competing models were evaluated using DeLong’s test.

Model calibration was assessed using calibration plots, calibration slope, calibration intercept, and the Brier score. Sensitivity, specificity, positive predictive value, and negative predictive value were calculated at the optimal probability threshold determined by the Youden index.

### Assessment of incremental predictive value

The additional prognostic value of the integrated biomarker model beyond conventional clinical variables was evaluated using continuous net reclassification improvement (NRI) and integrated discrimination improvement (IDI). These analyses were performed to determine whether the inclusion of biomarkers improved risk stratification compared with the clinical model alone.

### Internal validation and nomogram development

Internal validation was performed using bootstrap resampling with 1,000 iterations to estimate model optimism and obtain optimism-corrected performance measures. Bootstrap validation was used to assess the stability, discrimination, and calibration of the final model.

A prognostic nomogram was subsequently developed based on the final multivariable model to facilitate individualized estimation of 30-day mortality risk. Predictor scores were assigned proportionally according to the magnitude of the corresponding regression coefficients. The performance of the nomogram was evaluated using discrimination and calibration metrics derived from bootstrap validation.

### Ethical approval

The study was conducted in accordance with the ethical principles of the Declaration of Helsinki and was approved by the Institutional Review Board of Shandong Provincial Third Hospital (Approval No. KYLL-2026167). Given the retrospective observational design and use of anonymized clinical data, the requirement for written informed consent was waived by the ethics committee. All patient information was de-identified prior to analysis, and confidentiality was maintained throughout the study.

## Results

### Baseline characteristics

The baseline demographic, clinical, imaging, and treatment characteristics of the study population are summarized in Table 1. Data completeness was high for all variables, with a maximum of 3.2% missing for any individual variable (mean 1.8%; Supplementary Table S1). There were no significant differences in missingness patterns between survivors and no survivors (Supplementary Table S1). A total of 572 patients with acute aortic dissection (AAD) were included in the study. During the 30-day follow-up period, 130 patients (22.7%) died, whereas 442 patients (77.3%) survived. Compared with survivors, non-survivors were significantly older (70.57 ± 8.81 vs. 59.26 ± 9.70 years, *P* < 0.001) and exhibited a more adverse clinical profile at presentation. Non-survivors had significantly lower systolic blood pressure (109.27 ± 19.90 vs. 129.63 ± 19.64 mmHg, *P* < 0.001), higher heart rate (100.51 ± 18.27 vs. 86.98 ± 15.46 bpm, *P* < 0.001), and were more likely to present with diabetes mellitus (28.5% vs. 16.1%, *P* = 0.001), smoking history (49.2% vs. 32.4%, *P* = 0.001), coronary artery disease (29.2% vs. 12.9%, *P* < 0.001), and chronic kidney disease (20.0% vs. 4.5%, *P* < 0.001). Surgical intervention was performed less frequently among non-survivors (58.5% vs. 79.6%, *P* < 0.001), whereas medical management alone was more common in this group.

**Table 1:** Baseline characteristics of the study population.

Variable	Total (*N* = 572)	Survivors (*n* = 442)	30-day non-survivors (*n* = 130)	*P* value
Demographics				
Age (years), mean ± SD	61.83 ± 10.62	59.26 ± 9.70	70.57 ± 8.81	**<0** **.** **001**
Weight (kg), mean ± SD	75.30 ± 11.64	75.50 ± 11.80	74.61 ± 11.10	0.441
Height (cm), mean ± SD	170.40 ± 7.88	170.12 ± 7.94	171.32 ± 7.61	0.127
BMI (kg/m^2^), mean ± SD	26.08 ± 4.65	26.24 ± 4.68	25.56 ± 4.54	0.142
Male sex, *n* (%)	410 (71.7)	316 (71.5)	94 (72.3)	0.857
Medical History, *n* (%)				
Hypertension	289 (50.5)	221 (50.0)	68 (52.3)	0.642
Diabetes mellitus	108 (18.9)	71 (16.1)	37 (28.5)	**0** **.** **001**
Dyslipidemia	151 (26.4)	114 (25.8)	37 (28.5)	0.544
Smoking	207 (36.2)	143 (32.4)	64 (49.2)	**0** **.** **001**
Alcohol consumption	105 (18.4)	79 (17.9)	26 (20.0)	0.580
Coronary artery disease	95 (16.6)	57 (12.9)	38 (29.2)	**<0** **.** **001**
Prior stroke	52 (9.1)	36 (8.1)	16 (12.3)	0.147
Chronic kidney disease	46 (8.0)	20 (4.5)	26 (20.0)	**<0** **.** **001**
COPD	23 (4.0)	13 (2.9)	10 (7.7)	**0** **.** **015**
Marfan syndrome	18 (3.1)	12 (2.7)	6 (4.6)	0.275
Clinical Presentation				
Symptom-onset-to-admission (h), mean ± SD	12.26 ± 6.47	12.50 ± 6.52	11.43 ± 6.26	0.096
Admission time (h), mean ± SD	12.51 ± 6.73	12.52 ± 6.70	12.48 ± 6.87	0.956
Diagnostic delay (h), mean ± SD	12.79 ± 6.52	12.94 ± 6.44	12.30 ± 6.80	0.327
Chest pain, *n* (%)	474 (82.9)	365 (82.6)	109 (83.8)	0.736
Back pain, *n* (%)	260 (45.5)	205 (46.4)	55 (42.3)	0.412
Syncope, *n* (%)	61 (10.7)	43 (9.7)	18 (13.8)	0.180
Dyspnea, *n* (%)	166 (29.0)	132 (29.9)	34 (26.2)	0.413
Neurological deficit, *n* (%)	65 (11.4)	49 (11.1)	16 (12.3)	0.700
Shock, *n* (%)	90 (15.7)	67 (15.2)	23 (17.7)	0.486
Cardiac tamponade, *n* (%)	76 (13.3)	59 (13.3)	17 (13.1)	0.937
Vital Signs, mean ± SD				
Systolic blood pressure (mmHg)	125.00 ± 21.45	129.63 ± 19.64	109.27 ± 19.90	**<0** **.** **001**
Diastolic blood pressure (mmHg)	75.51 ± 12.21	75.89 ± 12.53	74.20 ± 11.00	0.165
Mean arterial pressure (mmHg)	92.01 ± 10.85	93.80 ± 10.59	85.89 ± 9.43	**<0** **.** **001**
Heart rate (beats/min)	90.05 ± 17.10	86.98 ± 15.46	100.51 ± 18.27	**<0** **.** **001**
Respiratory rate (breaths/min)	20.13 ± 4.03	20.26 ± 3.98	19.69 ± 4.19	0.157
SpO_2_ (%)	95.14 ± 3.1	95.07 ± 3.16	95.17 ± 2.98	0.738
Temperature (°C)	36.79 ± 0.52	36.80 ± 0.52	36.76 ± 0.50	0.548
Imaging findings, *n* (%)				
Stanford type A dissection	319 (55.8)	237 (53.6)	82 (63.1)	0.056
DeBakey type I	298 (52.1)	227 (51.4)	71 (54.6)	0.513
Aortic rupture	268 (46.9)	206 (46.6)	62 (47.7)	0.827
Malperfusion syndrome	257 (44.9)	204 (46.2)	53 (40.8)	0.278
Pericardial effusion	287 (50.0)	221 (50.0)	66 (50.8)	0.877
Pleural effusion	278 (48.6)	210 (47.5)	68 (52.3)	0.336
Patent false lumen	286 (50.0)	219 (49.5)	67 (51.5)	0.690
False lumen thrombosis	253 (44.2)	201 (45.5)	52 (40.0)	0.269
Treatment strategy, *n* (%)				
Surgical management alone	352 (61.5%)	312 (70.6%)	40 (30.8%)	<0.001
Endovascular intervention alone	54 (9.4%)	40 (9.0%)	14 (10.8%)	0.544
Combined surgical + endovascular	54 (9.4%)	40 (9.0%)	14 (10.8%)	0.544
Medical therapy alone	112 (19.6%)	50 (11.3%)	62 (47.7%)	<0.001

Continuous variables are presented as mean ± standard deviation (SD) and categorical variables as number (%). Percentages may not sum to 100% due to rounding. Comparisons were performed using Student’s *t*-test, Mann–Whitney *U* test, *χ*^2^ test, or Fisher’s exact test as appropriate. BMI, body mass index; COPD, chronic obstructive pulmonary disease. “Non-survivors are defined as patients who died within 30 days of hospital admission, consistent with the primary outcome definition.”.

Bold values indicate statistical significance (*P* < 0.05).

### Laboratory findings

Admission laboratory analyses demonstrated significant differences between survivors and non-survivors. Detailed laboratory results are presented in Table 2. Patients who died within 30 days had significantly higher concentrations of inflammatory markers, cardiac biomarkers, and coagulation-related parameters. Specifically, leukocytes (14.52 ± 4.90 vs. 10.78 ± 4.17 × 10^9^/L, *P* < 0.001), cTnI (0.46 ± 0.66 vs. 0.04 ± 0.06 ng/mL, *P* < 0.001), CK-MB (23.85 ± 11.01 vs. 17.60 ± 7.50 ng/mL, *P* < 0.001), BNP (656.09 ± 432.69 vs. 179.43 ± 127.34 pg/mL, *P* < 0.001), D-dimer (18.38 ± 10.04 vs. 5.57 ± 4.04 mg/L, *P* < 0.001), FDP (13.98 ± 8.14 vs. 7.97 ± 5.06 μg/mL, *P* < 0.001), serum creatinine (1.56 ± 0.57 vs. 1.11 ± 0.41 mg/dL, *P* < 0.001), and lactate (3.29 ± 1.84 vs. 1.82 ± 0.80 mmol/L, *P* < 0.001) were significantly elevated in non-survivors compared with survivors. In contrast, platelet counts and albumin levels (21.53 ± 9.48 vs. 29.68 ± 9.39 g/L, *P* < 0.001) were significantly lower among non-survivors. All inflammatory biomarkers including CRP, procalcitonin, ESR, IL-6, TNF-α, NLR, PLR, and SII were significantly elevated in non-survivors (all *P* < 0.001). These findings suggest that enhanced myocardial injury, hemodynamic stress, coagulation activation, and organ dysfunction are associated with increased short-term mortality risk in patients with AAD.

**Table 2:** Laboratory characteristics according to 30-day mortality.

Variable	Total (*N* = 572)	Survivors (*n* = 442)	30-day non-survivors (*n* = 130)	*P* value
Hemoglobin (g/dL)	13.54 ± 1.80	13.51 ± 1.81	13.67 ± 1.76	0.359
Hematocrit (%)	40.15 ± 4.98	40.12 ± 4.93	40.25 ± 5.17	0.791
Leukocytes (×10^9^/L)	11.63 ± 4.62	10.78 ± 4.17	14.52 ± 4.90	**<0** **.** **001**
Platelets (×10^9^/L)	239.1 ± 80.25	250.25 ± 77.08	225.75 ± 76.17	**<0** **.** **001**
RDW (%)	10.01 ± 2.78	10.08 ± 2.72	9.79 ± 2.96	0.296
CRP (mg/L)	30.70 ± 10.94	30.02 ± 1.52	60.00 ± 10.36	**<0** **.** **001**
Procalcitonin (ng/mL)	3.64 ± 1.92	2.93 ± 1.41	6.04 ± 1.43	**<0** **.** **001**
ESR (mm/h)	30.79 ± 2.10	30.04 ± 1.56	60.35 ± 1.66	**<0** **.** **001**
IL-6 (pg/mL)	3.95 ± 2.23	3.22 ± 1.82	6.45 ± 1.58	**<0** **.** **001**
TNF-α (pg/mL)	3.83 ± 2.05	3.15 ± 1.60	6.13 ± 1.70	**<0** **.** **001**
NLR	3.26 ± 1.61	3.02 ± 1.57	4.06 ± 1.49	**<0** **.** **001**
MLR	2.95 ± 1.69	2.96 ± 1.68	2.94 ± 1.76	0.903
PLR	3.35 ± 1.69	3.11 ± 1.60	4.15 ± 1.76	**<0** **.** **001**
SII	3.32 ± 1.64	3.06 ± 1.58	4.22 ± 1.52	**<0** **.** **001**
cTnI (ng/mL)	0.14 ± 0.36	0.04 ± 0.06	0.46 ± 0.66	**<0** **.** **001**
CK-MB (ng/mL)	19.02 ± 8.81	17.60 ± 7.50	23.85 ± 11.01	**<0** **.** **001**
BNP (pg/mL)	287.76 ± 307.88	179.43 ± 127.34	656.09 ± 432.69	**<0** **.** **001**
NT-proBNP (pg/mL)	431.64 ± 461.82	269.14 ± 191.01	984.13 ± 649.03	**<0** **.** **001**
Myoglobin (ng/mL)	121.82 ± 66.65	99.83 ± 53.77	196.58 ± 49.92	**<0** **.** **001**
Troponin (ng/mL)	0.17 ± 0.43	0.05 ± 0.07	0.55 ± 0.79	**<0** **.** **001**
LDH (U/L)	307.58 ± 106.80	292.42 ± 103.16	359.14 ± 103.17	**<0** **.** **001**
D-dimer (mg/L)	8.48 ± 8.01	5.57 ± 4.04	18.38 ± 10.04	**<0** **.** **001**
FDP (μg/mL)	9.33 ± 6.41	7.97 ± 5.06	13.98 ± 8.14	**<0** **.** **001**
PT ratio	1.05 ± 0.20	1.00 ± 0.18	1.20 ± 0.19	**<0** **.** **001**
INR	1.03 ± 0.21	0.97 ± 0.19	1.22 ± 0.17	**<0** **.** **001**
APTT ratio	1.01 ± 0.20	1.01 ± 0.21	1.02 ± 0.20	0.516
Fibrinogen (g/L)	3.35 ± 0.36	3.51 ± 0.20	2.80 ± 0.22	**<0** **.** **001**
Antithrombin (%)	1.01 ± 0.19	1.00 ± 0.19	1.03 ± 0.19	0.116
Creatinine (mg/dL)	1.21 ± 0.49	1.11 ± 0.41	1.56 ± 0.57	**<0** **.** **001**
Urea (mg/dL)	35.38 ± 12.06	35.29 ± 11.71	35.69 ± 13.23	0.740
eGFR (mL/min/1.73m^2^)	76.78 ± 25.59	82.75 ± 22.31	56.50 ± 25.68	**<0** **.** **001**
ALT (U/L)	34.67 ± 12.98	30.32 ± 10.16	49.46 ± 10.41	**<0** **.** **001**
AST (U/L)	34.34 ± 12.68	30.02 ± 9.82	49.05 ± 10.00	**<0** **.** **001**
Bilirubin (μmol/L)	33.25 ± 10.41	30.63 ± 8.97	42.17 ± 10.05	**<0** **.** **001**
Albumin (g/L)	27.83 ± 10.00	29.68 ± 9.39	21.53 ± 9.48	**<0** **.** **001**
Glucose (mg/dL)	119.99 ± 29.71	120.70 ± 29.61	117.58 ± 30.05	0.294
Lactate (mmol/L)	2.16 ± 1.28	1.82 ± 0.80	3.29 ± 1.84	**<0** **.** **001**

Values are presented as mean ± SD. *P* values were obtained using Student’s *t*-test or Mann–Whitney *U* test according to data distribution. RDW, red cell distribution width; CRP, C-reactive protein; ESR, erythrocyte sedimentation rate; NLR, neutrophil-to-lymphocyte ratio; PLR, platelet-to-lymphocyte ratio; SII, systemic immune-inflammation index; cTnI, cardiac troponin I; BNP, B-type natriuretic peptide; FDP, fibrin/fibrinogen degradation products.

Bold values indicate statistical significance (*P* < 0.05).

### Univariable and multivariable predictors of 30-day mortality

Univariable logistic regression identified several factors significantly associated with 30-day mortality, including advanced age (OR = 1.14, 95% CI: 1.11–1.17, *P* < 0.001), Stanford type A dissection (OR = 1.48, 95% CI: 0.99–2.21, *P* = 0.057), lower systolic blood pressure (OR = 0.95, 95% CI: 0.94–0.96, *P* < 0.001), elevated serum creatinine (OR = 8.17, 95% CI: 4.98–13.41, *P* < 0.001), absence of surgical intervention (OR = 0.20, 95% CI: 0.13–0.30, *P* < 0.001), elevated cTnI (OR = 3.00, 95% CI: 1.92–4.67, *P* < 0.001), elevated BNP (OR = 3.39, 95% CI: 1.84–6.24, *P* < 0.001), elevated D-dimer (OR = 3.57, 95% CI: 1.26–10.12, *P* = 0.017), and elevated FDP (OR = 2.90, 95% CI: 1.66–5.10, *P* < 0.001).

In the multivariable logistic regression model adjusted for age, Stanford classification, symptom-onset-to-admission time, systolic blood pressure, creatinine, treatment strategy, and all biomarkers, age (adjusted OR = 1.16, 95% CI: 1.12–1.21, *P* < 0.001), symptom-onset-to-admission time (adjusted OR = 0.93, 95% CI: 0.89–0.98, *P* = 0.010), systolic blood pressure (adjusted OR = 0.94, 95% CI: 0.92–0.96, *P* < 0.001), surgical management (adjusted OR = 0.13, 95% CI: 0.06–0.25, *P* < 0.001), elevated cTnI (adjusted OR = 3.76, 95% CI: 1.87–7.56, *P* < 0.001), and elevated BNP remained independently associated with 30-day mortality. Increasing age and serum creatinine levels were associated with a higher risk of death, whereas higher systolic blood pressure and surgical treatment were associated with improved survival. Among biomarkers, elevated cTnI and BNP demonstrated independent prognostic significance after adjustment for clinical covariates. In contrast, CK-MB (adjusted OR = 1.19, 95% CI: 0.27–5.25, *P* = 0.823) and FDP (adjusted OR = 1.63, 95% CI: 0.73–3.62, *P* = 0.231) were not independently associated with mortality after multivariable adjustment. Correlation analysis revealed only weak correlations between CK-MB and cTnI (*ρ* = 0.104, *P* = 0.013) and between FDP and D-dimer (*ρ* = 0.191, *P* < 0.001), (Supplementary Table S3), indicating that multicollinearity does not explain the loss of significance. Rather, these findings suggest that CK-MB and FDP provide limited additional prognostic information beyond cTnI and D-dimer, respectively, consistent with their lower tissue specificity and signal strength in the setting of acute aortic dissection. D-dimer remained independently associated with 30-day mortality after multivariable adjustment (adjusted OR: 2.70, 95% CI: 1.17–6.24; *P* = 0.020). The results of the univariable and multivariable analyses are summarized in Table 3.

**Table 3:** Univariate and multivariable logistic regression analysis for 30-day mortality.

Variable	Unadjusted OR (95% CI)	*P* value	Adjusted OR (95% CI)	*P* value
Age	1.14 (1.11–1.17)	<0.001	1.16 (1.12–1.21)	<0.001
Stanford type A	1.48 (0.99–2.21)	0.057	1.67 (0.89–3.14)	0.111
Symptom-onset-to-admission time	0.97 (0.95–1.00)	0.096	0.93 (0.89–0.98)	0.010
Systolic blood pressure	0.95 (0.94–0.96)	<0.001	0.94 (0.92–0.96)	<0.001
Creatinine	8.17 (4.98–13.41)	<0.001	13.26 (5.96–29.53)	<0.001
Surgical management	0.20 (0.13–0.30)	<0.001	0.13 (0.06–0.25)	<0.001
Elevated cTnI	3.00 (1.92–4.67)	<0.001	3.76 (1.87–7.56)	<0.001
Elevated CK-MB	1.10 (0.47–2.59)	0.829	1.19 (0.27–5.25)	0.823
Elevated BNP	3.39 (1.84–6.24)	<0.001	6.59 (2.76–15.76)	<0.001
Elevated D-dimer	3.57 (1.26–10.12)	0.017	2.70 (1.17–6.24)	0.020
Elevated FDP	2.90 (1.66–5.10)	<0.001	1.63 (0.73–3.62)	0.231

Multivariable logistic regression included age, Stanford classification, symptom-onset-to-admission time, systolic blood pressure, creatinine, treatment strategy, cTnI, CK-MB, BNP, D-dimer, and FDP. OR, odds ratio; CI, confidence interval; cTnI, cardiac troponin I; CK-MB, creatine kinase-MB; BNP, B-type natriuretic peptide; FDP, fibrin/fibrinogen degradation products. Cutoffs: cTnI > 0.04 ng/mL, CK-MB > 5.0 ng/mL, BNP > 100 pg/mL, D-dimer > 0.5 mg/L, FDP >5.0 μg/mL.

### Discriminative performance of individual biomarkers and prediction models

**Figure 2:**
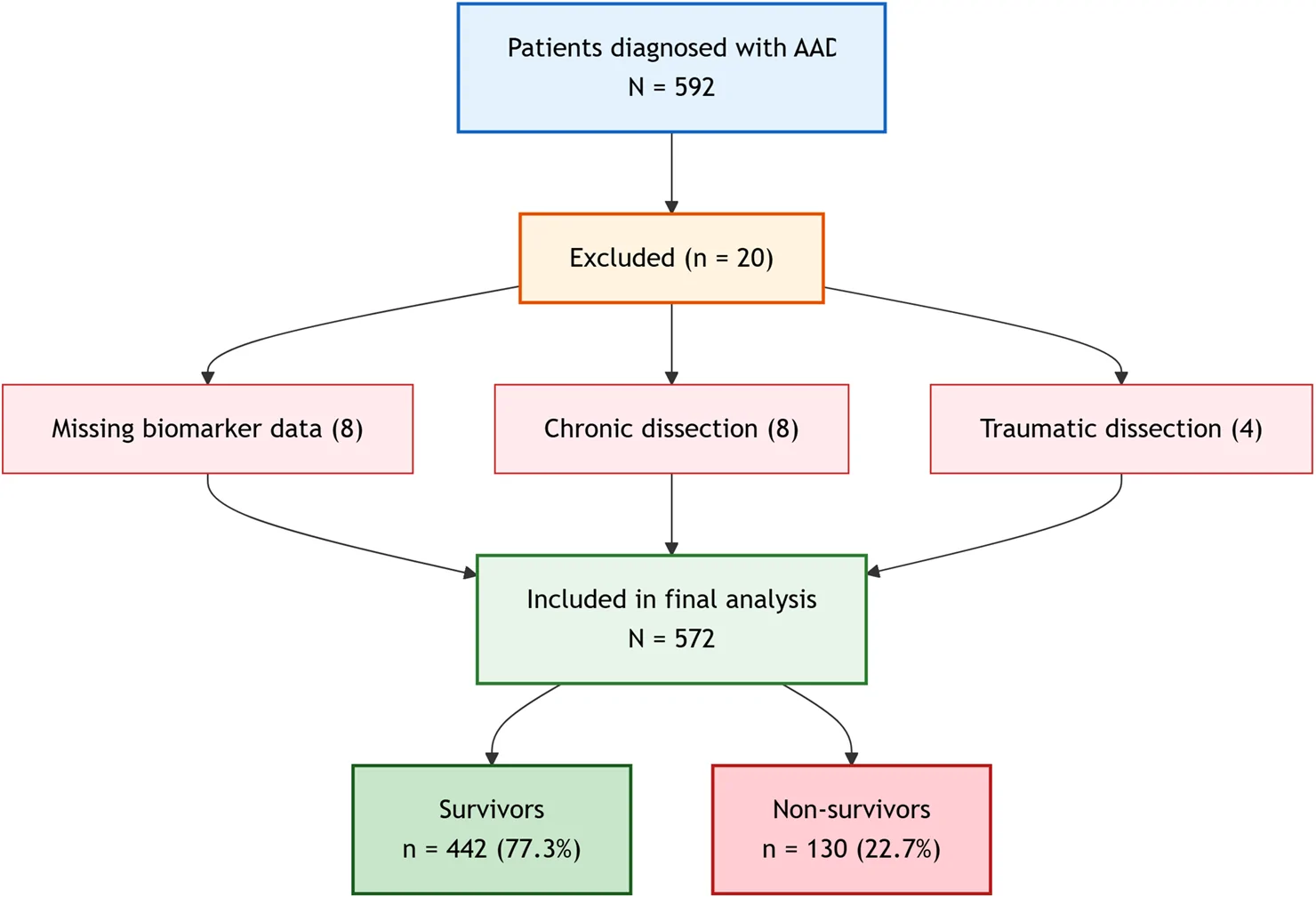
Receiver operating characteristic (ROC) curves for individual biomarkers and the integrated model for predicting 30-day mortality in patients with acute aortic dissection.

Receiver operating characteristic (ROC) curve analyses were performed to evaluate the prognostic performance of individual biomarkers and combined prediction models (Figure 2). Among the individual biomarkers, cTnI demonstrated the highest discriminative ability (AUC = 0.623, 95% CI: 0.579–0.666), followed by BNP (AUC = 0.587, 95% CI: 0.554–0.620) and D-dimer (AUC = 0.536, 95% CI: 0.515–0.556). However, the predictive performance of each biomarker alone was modest.

Combining biomarkers resulted in improved discrimination compared with individual biomarker models. The cTnI + BNP combination achieved an apparent AUC of 0.672 (95% CI: 0.626–0.717), cTnI + Ddimer achieved 0.651 (95% CI: 0.607–0.695), and BNP + Ddimer achieved 0.614 (95% CI: 0.577–0.650). The clinical model comprising age, Stanford classification, SBP, creatinine, and symptom on set to admission time achieved an apparent AUC of 0.887 (optimism corrected AUC = 0.84). The integrated model incorporating clinical variables and all biomarkers demonstrated good predictive performance, with an apparent AUC of 0.946 (95% CI: 0.925–0.967) and an optimism corrected AUC of 0.89 (95% CI: 0.83–0.93). The integrated model demonstrated improved discrimination compared with the clinical model (DeLong’s test *P* = 0.002 for apparent AUC comparison; optimism corrected comparison also favored the integrated model). The optimism corrected AUC improvement of 0.05 represents a modest but statistically significant increment, and the NRI (0.32, *P* < 0.01) and IDI (0.11, *P* < 0.01) further confirm meaningful risk reclassification beyond clinical variables alone. The integrated model also significantly outperformed cTnI alone, BNP alone, D-dimer alone, and all two-biomarker combinations (all *P* < 0.001). At the optimal cutoff determined by the Youden index, the integrated model achieved a sensitivity of 76.2%, specificity of 96.8%, positive predictive value of 87.6%, and negative predictive value of 93.2%. The ROC analysis results are presented in Table 4 and Figure 2.

**Table 4:** Discriminative performance of individual biomarkers and prediction models.

Model	Apparent AUC (95% CI)	Optimism-corrected AUC	Sensitivity (%)	Specificity (%)	PPV (%)	NPV (%)	*P* value*
Clinical model	0.887 (0.858–0.916)	0.84	63.1	95.5	80.4	89.8	Reference
cTnI alone	0.623 (0.579–0.666)	0.61	51.3	72.4	35.8	83.1	<0.001
BNP alone	0.587 (0.554–0.620)	0.57	48.5	69.2	31.2	82.3	<0.001
D-dimer alone	0.536 (0.515–0.556)	0.52	42.3	64.8	26.4	79.6	<0.001
cTnI + BNP	0.672 (0.626–0.717)	0.65	58.5	75.9	42.1	85.9	<0.001
cTnI + D-dimer	0.651 (0.607–0.695)	0.63	55.4	73.6	38.7	84.8	<0.001
BNP + D-dimer	0.614 (0.577–0.650)	0.60	50.8	71.2	34.5	83.1	<0.001
**Integrated model**	**0.946** **(0.925–0.967)**	**0** **.** **89**	**76** **.** **2**	**96** **.** **8**	**87** **.** **6**	**93** **.** **2**	**0** **.** **002**

* AUC indicates area under the receiver-operating-characteristic curve. PPV, positive predictive value; NPV, negative predictive value. Compared with the clinical model using DeLong’s test. Sensitivity, specificity, PPV, and NPV for all models were calculated at the Youden-index-derived optimal cutoff. cTnI, cardiac troponin I; BNP, B-type natriuretic peptide; CI, confidence interval. Optimism-corrected AUCs were derived from bootstrap internal validation with 1,000 resamples.

Bold values indicate statistical significance (*P* < 0.05).

### Nomogram for predicting 30-day mortality

**Figure 3:**
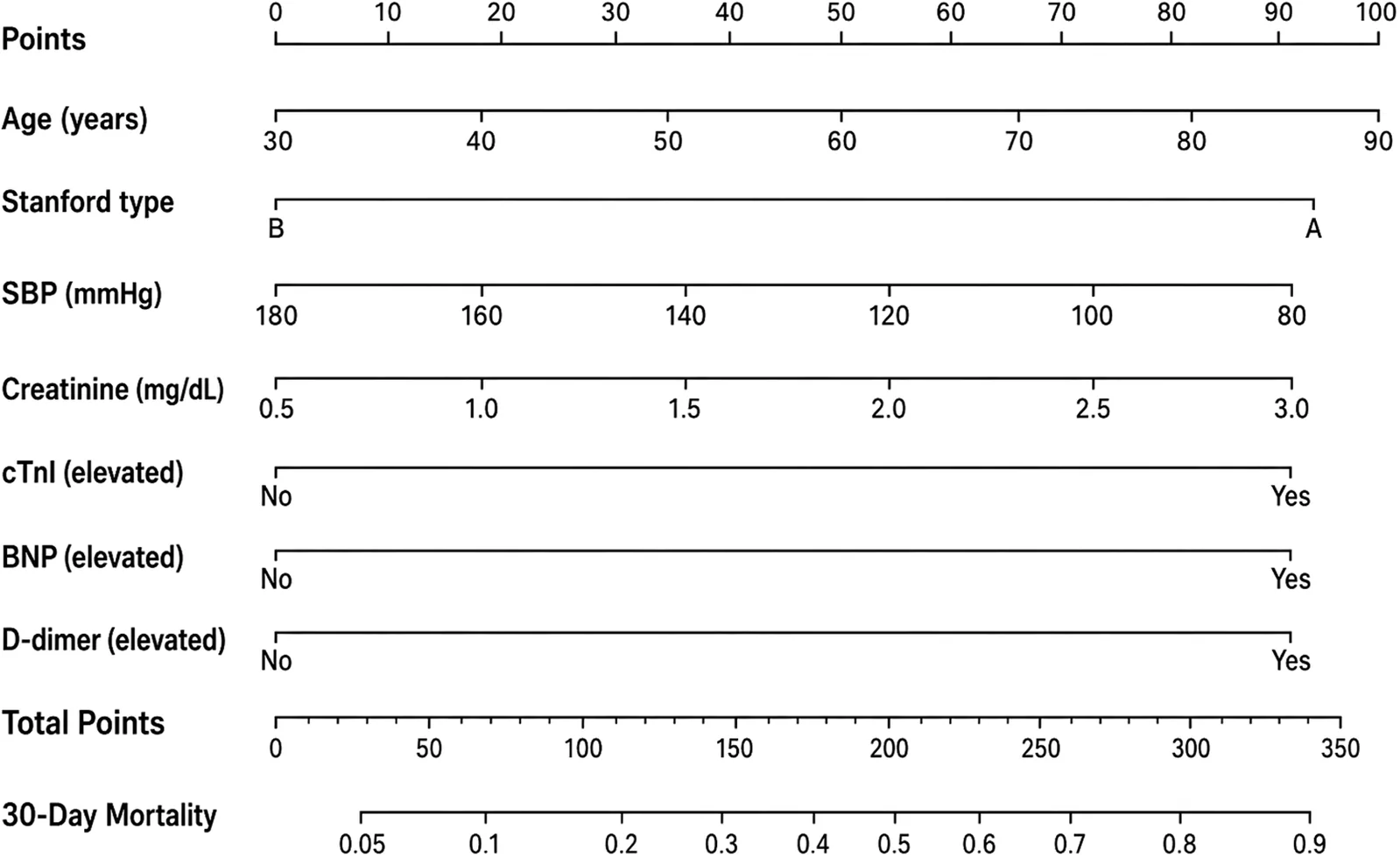
Nomogram for predicting 30-day mortality in patients with acute aortic dissection. The nomogram incorporates seven independent predictors: age, Stanford classification, systolic blood pressure, creatinine, cTnI (elevated >0.04 ng/mL), BNP (elevated >100 pg/mL), and D-dimer (elevated >0.5 mg/L). To use the nomogram, locate each variable on its corresponding axis, draw a vertical line to the “Points” axis to obtain the score for each variable, sum all scores to obtain “Total Points,” and draw a vertical line from the “Total Points” axis to the “30-Day Mortality” axis to determine the predicted probability. The nomogram demonstrated excellent discrimination with a concordance index of 0.89 (95% CI: 0.83–0.93). cTnI, cardiac troponin I; BNP, B-type natriuretic peptide; SBP, systolic blood pressure; CI, confidence interval.

Based on the final multivariable model, a parsimonious prognostic nomogram was developed incorporating the independent predictors available at presentation: age, Stanford classification, systolic blood pressure, creatinine, cTnI (elevated), BNP (elevated), and Ddimer (elevated) (Figure 3). Treatment strategy was deliberately excluded from the nomogram because it is determined after risk stratification and is strongly influenced by clinical severity, which would limit the tool’s utility for pretreatment decision-making. Each predictor was assigned a point score based on its regression coefficient: age (30–90 years: 0–90 points), Stanford type (B: 0, A: 15 points), systolic blood pressure (180–80 mmHg: 0–70 points), creatinine (0.5–3.0 mg/dL: 0–100 points), cTnI (No: 0, Yes: 55 points), BNP (No: 0, Yes: 75 points), and Ddimer (No: 0, Yes: 40 points). Each predictor was assigned a point score based on its regression coefficient: age (30–90 years: 0–90 points), Stanford type (B: 0, A: 15 points), systolic blood pressure (180–80 mmHg: 0–70 points), creatinine (0.5–3.0 mg/dL: 0–100 points), cTnI (No: 0, Yes: 55 points), BNP (No: 0, Yes: 75 points), and D-dimer (No: 0, Yes: 40 points). The total points are summed and matched to the corresponding 30-day mortality probability, ranging from 0.05 (0 points) to 0.90 (350 points). The nomogram demonstrated excellent discrimination with a concordance index of 0.89 (95% CI: 0.83–0.93) after internal validation, consistent with the integrated model performance. The nomogram provides a practical bedside tool for rapid risk stratification in emergency settings.

**Example**: A 70-year-old patient (40 points) with Type A dissection (15 points), SBP 100 mmHg (50 points), creatinine 2.0 mg/dL (60 points), elevated cTnI (55 points), elevated BNP (75 points), and elevated D-dimer (40 points) has total points = 335, corresponding to an estimated 30-day mortality risk of approximately 85%.

### Incremental predictive value of the integrated biomarker model

The addition of biomarkers to the clinical prediction model resulted in significant improvements in risk stratification. Compared with the clinical model alone, the integrated biomarker model demonstrated a continuous net reclassification improvement (NRI) of 0.32 (*P* < 0.01) and an integrated discrimination improvement (IDI) of 0.11 (*P* < 0.01), indicating enhanced classification and discrimination of patients at risk of 30-day mortality. These findings suggest that the incorporation of biomarker information provides incremental prognostic value beyond conventional clinical predictors. Detailed reclassification statistics are shown in Table 5A.

**Table 5A:** Reclassification performance.

Comparison	NRI	*P* value	IDI	*P* value
Integrated model vs. clinical model	**0** **.** **32**	**<0** **.** **01**	**0** **.** **11**	**<0** **.** **01**

NRI, net reclassification improvement; IDI, integrated discrimination improvement.

Bold values indicate statistical significance (*P* < 0.05).

### Internal validation and calibration

Internal validation using bootstrap resampling with 1,000 iterations demonstrated robust model performance with modest optimism. The optimism-corrected AUC was 0.89 (95% CI: 0.83–0.93), confirming good discriminative ability after accounting for overfitting, and remained superior to the clinical model’s optimism-corrected AUC of 0.84. The optimism estimate of 0.056 indicates that the apparent performance is slightly inflated, as expected in prediction model development. Calibration analysis revealed close agreement between predicted and observed mortality risks, with a calibration slope of 1.02 (95% CI: 0.85–1.19) and calibration intercept of 0.01 (95% CI: −0.15 to 0.17), indicating excellent model calibration. The Brier score of 0.068 (95% CI: 0.055–0.081) reflected good overall predictive accuracy. The optimism-corrected performance measures confirmed the stability and reliability of the integrated prediction model. Internal validation results are summarized in Table 5B.

**Table 5B:** Internal validation metrics.

Metric	Value
Bootstrap resamples	1,000
Optimism-corrected AUC	**0** **.** **89**
Calibration slope	1.02
Calibration intercept	0.01
Brier score	**0** **.** **068**

Bold values indicate statistical significance (*P* < 0.05).

### Performance of the final integrated prediction model

The final integrated prediction model exhibited excellent discrimination and calibration for predicting 30-day mortality in patients with AAD. At the optimal probability threshold, the model achieved high sensitivity (76.2%, 95% CI: 68.7–82.4) and specificity (96.8%, 95% CI: 95.0–98.1), with favorable positive predictive value (87.6%, 95% CI: 80.2–92.5) and negative predictive value (93.2%, 95% CI: 90.6–95.2). The model also demonstrated a low Brier score of 0.068 (95% CI: 0.055–0.081), indicating accurate estimation of individual mortality risk. These findings support the clinical utility of the integrated model as a practical tool for early risk stratification and individualized prognostic assessment in patients presenting with acute aortic dissection. The detailed performance metrics of the final model are presented in Table 6.

**Table 6:** Final integrated model performance metrics.

Performance metric	Estimate	95% Confidence interval
Area under the curve (AUC)	0.946	0.925–0.967
Optimism-corrected AUC	0.89	0.83–0.93
Optimism (Δ)	0.056	—
Sensitivity (%)	76.2	68.7–82.4
Specificity (%)	96.8	95.0–98.1
Positive predictive value (%)	87.6	80.2–92.5
Negative predictive value (%)	93.2	90.6–95.2
Brier score	0.068	0.055–0.081
Calibration slope^†^	1.02	0.85–1.19
Calibration intercept^†^	0.01	−0.15 to 0.17

† Calibration statistics were derived from bootstrap resampling (1,000 iterations). AUC, area under the receiver-operating-characteristic curve; PPV, positive predictive value; NPV, negative predictive value.

Model performance was assessed using discrimination, calibration, and predictive accuracy measures. Confidence intervals were calculated by bootstrap resampling where applicable.

## Discussion

In this retrospective cohort study of 572 patients with AAD, we developed and internally validated an integrated prognostic model incorporating routinely available clinical variables and admission biomarkers for the prediction of 30-day mortality. The principal findings were threefold. First, elevated cTnI and BNP were independently associated with short-term mortality after adjustment for established clinical risk factors. Second, the integration of biomarker information provided statistically significant incremental prognostic value over conventional clinical assessment alone, with an optimism corrected AUC improvement of 0.05 (from 0.84 to 0.89) and significant NRI (0.32, *P* < 0.01) and IDI (0.11, *P* < 0.01) values, though the clinical utility of this modest improvement requires external validation. Third, the resulting nomogram, incorporating age, Stanford classification, systolic blood pressure, serum creatinine, cTnI, BNP, and D-dimer, provided a practical tool for individualized bedside risk stratification. Collectively, these findings support the value of a multi marker strategy that captures complementary biological pathways involved in AAD progression and adverse outcomes.

The prognostic assessment of patients with AAD remains challenging because mortality is influenced by multiple interconnected mechanisms, including aortic rupture, malperfusion, myocardial injury, hemodynamic instability, and systemic inflammatory and coagulation responses (9). Existing risk stratification approaches rely largely on clinical presentation, imaging findings, and procedural factors (17–20). Although several biomarkers have been proposed as prognostic indicators, most studies have evaluated single biomarkers in isolation (21). In contrast, the present study evaluated a biologically complementary panel of biomarkers representing myocardial injury, hemodynamic stress, and coagulation activation within a unified prognostic framework. The observed improvement in model performance, including an optimism-corrected AUC of 0.89 and significant NRI and IDI values, suggests that integrating information from multiple pathophysiological domains provides a more comprehensive assessment of mortality risk. However, the difference between the apparent AUC (0.946) and corrected AUC (0.89) indicates some degree of overfitting, underscoring the need for external validation.

Among the evaluated biomarkers, cTnI emerged as an independent predictor of 30-day mortality. This finding is consistent with previous investigations demonstrating that myocardial injury is associated with poor outcomes in AAD (22). Elevated troponin levels in this setting may reflect coronary malperfusion resulting from extension of the dissection flap into the coronary ostia, myocardial ischemia caused by compromised coronary blood flow, severe aortic regurgitation, or global hypoperfusion associated with cardiogenic or obstructive shock (23). Consequently, cTnI likely serves as an integrated marker of cardiovascular injury and disease severity (24). Importantly, cTnI remained independently associated with mortality after adjustment for clinical variables and other biomarkers, underscoring its prognostic relevance beyond its traditional diagnostic role.

BNP demonstrated the strongest biomarker association with mortality in the multivariable model. Elevated BNP concentrations likely reflect the substantial hemodynamic burden imposed by acute aortic dissection, including left ventricular pressure overload, acute aortic regurgitation, ventricular dysfunction, and neurohormonal activation (25). Previous studies have reported associations between BNP and adverse outcomes across a broad range of acute cardiovascular conditions, including acute heart failure, acute coronary syndromes, and acute aortic syndromes (26). Our findings extend this evidence by demonstrating that BNP provides independent prognostic information in AAD and contributes substantially to risk discrimination when incorporated into a multivariable model. Notably, BNP exhibited a stronger adjusted association with mortality than cTnI, highlighting the importance of hemodynamic stress as a determinant of early adverse outcomes.

D-dimer remained independently associated with 30-day mortality after multivariable adjustment (adjusted OR: 2.70, 95% CI: 1.17–6.24; *P* = 0.020), although its effect size was smaller than that of cTnI (OR: 3.76) and BNP (OR: 6.59). This finding is consistent with D-dimer’s role as a marker of coagulation activation and disease extent. When considered alongside markers of organ dysfunction, hemodynamic compromise, and myocardial injury, D-dimer’s independent prognostic contribution is modest but significant. The persistence of D-dimer as an independent predictor after multivariable adjustment suggests that coagulation activation contributes to mortality risk even after accounting for other pathways (27). Elevated D-dimer concentrations are strongly associated with the presence of acute aortic syndromes and have become an important adjunctive diagnostic biomarker. However, when considered alongside markers of organ dysfunction, hemodynamic compromise, and myocardial injury, its independent prognostic contribution appears attenuated. Nevertheless, D-dimer was retained in the integrated prediction model and nomogram because of its established clinical relevance, widespread availability, and independent prognostic contribution (OR: 2.70, *P* = 0.020). These findings suggest that D-dimer provides complementary prognostic information beyond that captured by cTnI and BNP.

An important finding of the present study was the loss of prognostic significance of CK-MB and FDP after multivariable adjustment. Although both biomarkers were significantly elevated among non-survivors and demonstrated associations with mortality in unadjusted analyses, neither contributed independent prognostic information when cTnI, BNP, and clinical covariates were considered simultaneously (28). Correlation analysis revealed only weak correlations between CK-MB and cTnI (*ρ* = 0.104, *P* = 0.013) and between FDP and D-dimer (*ρ* = 0.191, *P* < 0.001), indicating that multicollinearity does not explain this finding. Rather, CK-MB likely provides less specific information than cTnI, which is the preferred marker of myocardial injury due to its higher cardiac specificity and sensitivity (29). Similarly, FDP reflects fibrinolytic activity that is less specific than D-dimer for the hypercoagulable state in acute aortic dissection. These observations support the use of a parsimonious biomarker strategy focused on cTnI, BNP, and D-dimer, rather than expanding prognostic models with additional biomarkers that provide limited incremental value.

Beyond identifying individual prognostic markers, our study demonstrates the value of an integrated biomarker approach. While the IRAD score relies solely on clinical presentation variables (30, 31), our model integrates admission biomarkers to capture complementary biological pathways, which may provide enhanced risk discrimination. The combined model significantly outperformed individual biomarkers and the clinical model alone, while reclassification analyses confirmed statistically significant improvements in patient risk categorization (NRI: 0.32, IDI: 0.11). However, head-to-head comparison within the same cohort is required to establish the relative predictive performance of our integrated model compared with established risk scores. These findings align with the broader cardiovascular literature, where multimarker strategies consistently outperform single-biomarker approaches because they capture distinct biological pathways contributing to disease progression. In AAD, where mortality is determined by a complex interaction of anatomical, hemodynamic, inflammatory, and ischemic processes, a multidimensional biomarker framework appears particularly appropriate.”

The nomogram developed from the final model may have practical clinical implications. Because all included variables are routinely available during the initial evaluation of patients with AAD, the nomogram can be readily implemented without specialized testing or substantial additional cost. Early identification of high-risk patients may facilitate timely referral to specialized centers, prioritization of intensive monitoring, optimization of perioperative management, and informed communication with patients and families regarding prognosis (20, 32). The discriminative performance of individual biomarkers in our study was modest, consistent with previous reports. Liu et al. reported an AUC of 0.65 for cTnI in predicting mortality, comparable to our finding of 0.623. This modest performance of single biomarkers underscores the rationale for a multimarker strategy, as no single biological pathway captured by a single marker is sufficient for comprehensive risk stratification (33, 34). Although the nomogram requires external validation before widespread adoption, its strong discrimination and favorable calibration suggest potential utility as a bedside decision-support tool. Established risk stratification tools, such as the Penn classification (35) and GERAADA score (36), rely primarily on anatomical, surgical, and organ malperfusion parameters without incorporating circulating biomarkers. The inclusion of cTnI, BNP, and D-dimer in our model addresses underlying myocardial, hemodynamic, and coagulation pathways, which may provide additional prognostic information. However, because direct head-to-head reconstruction of these scores in our cohort was not performed, formal comparative validation in external cohorts is necessary to determine the relative performance of our integrated model vs. these established tools.”

This study has several notable strengths. First, it included a relatively large cohort of patients with confirmed AAD and complete ascertainment of the primary outcome. Second, the analysis incorporated a comprehensive assessment of clinically relevant biomarkers representing distinct biological mechanisms implicated in AAD progression. Third, the study followed a rigorous analytical framework consistent with STROBE and TRIPOD recommendations, including multivariable modeling, assessment of discrimination and calibration, reclassification analyses, and bootstrap-based internal validation. Fourth, the final model was developed using biomarkers that are routinely available in contemporary clinical practice, enhancing its potential translational applicability.

Several limitations should be acknowledged. First, the retrospective single-center design may limit the generalizability of the findings and introduces the potential for residual confounding despite multivariable adjustment. Second, the study population was derived from a single healthcare system and consisted predominantly of Han Chinese patients; therefore, the model should be validated in geographically and ethnically diverse populations before clinical implementation. Third, biomarker measurements were obtained only at admission, and the potential prognostic value of serial biomarker assessment was not evaluated. Fourth, treatment allocation was not randomized and may have been influenced by clinical severity, introducing the possibility of confounding by indication. Fifth, although internal validation demonstrated robust model performance with modest optimism (0.056), external validation remains necessary to confirm reproducibility and transportability. Sixth, our comparison with established risk scores such as IRAD, Penn classification, and GERAADA was indirect; formal head-to-head validation in the same cohort is required to establish comparative performance and determine whether the integrated model provides superior risk stratification. Finally, the model does not incorporate all potentially relevant prognostic factors, including detailed genetic information, medication use, and advanced imaging-derived parameters.

## Conclusions

An integrated prognostic model incorporating age, Stanford classification, systolic blood pressure, serum creatinine, cTnI, BNP, and D-dimer demonstrated good discrimination (optimism-corrected AUC: 0.89) and significant incremental value over clinical variables alone (NRI = 0.32, IDI = 0.11). Among the evaluated biomarkers, cTnI (OR: 3.76), BNP (OR: 6.59), and D-dimer (OR: 2.70) emerged as independent predictors of 30-day mortality. However, the modest AUC improvement (ΔAUC = 0.05) and lack of head-to-head comparison with established risk scores (IRAD, Penn, GERAADA) warrant cautious interpretation and external validation before clinical implementation.

## Data Availability

The raw data supporting the conclusions of this article will be made available by the authors, without undue reservation.
