# Supplementary material for: Integrated biomarker model based on cTnI, BNP, and D-dimer for early risk stratification in acute aortic dissection
Source: Front Cardiovasc Med. 2026 Aug 27;13:1926181. doi: 10.3389/fcvm.2026.1926181 (PMC13558106; doi:10.3389/fcvm.2026.1926181)
Supplement: Supplementary file 1 [file Datasheet1.docx]

| **Supplementary Table S1. Per-Variable Missingness in the Study Cohort** | |
| --- | --- |
| **Variable** | **Missing n (%)** |
| Age | 0 (0%) |
| Sex | 0 (0%) |
| BMI | 8 (1.4%) |
| Hypertension | 0 (0%) |
| Diabetes mellitus | 0 (0%) |
| Smoking | 0 (0%) |
| Coronary artery disease | 0 (0%) |
| Chronic kidney disease | 0 (0%) |
| Symptom-onset-to-admission time | 5 (0.9%) |
| Systolic blood pressure | 0 (0%) |
| Diastolic blood pressure | 0 (0%) |
| Heart rate | 0 (0%) |
| Stanford classification | 0 (0%) |
| Creatinine | 0 (0%) |
| cTnI | 0 (0%) |
| CK-MB | 18 (3.1%) |
| BNP | 0 (0%) |
| D-dimer | 0 (0%) |
| FDP | 12 (2.1%) |
| Treatment strategy | 0 (0%) |
| *Data are presented as number of missing observations with percentage of total cohort (N = 572) in parentheses. Missing data rates were minimal across all variables (range: 0% to 3.2%; mean: 1.8%). There were no missing data for the primary outcome (30-day mortality) or for key biomarkers including cTnI, BNP, and D-dimer. Complete-case analysis was performed for the primary analyses given the low overall missingness (<5%). Abbreviations: BMI, body mass index; CAD, coronary artery disease; CKD, chronic kidney disease; COPD, chronic obstructive pulmonary disease; cTnI, cardiac troponin I; CK-MB, creatine kinase-MB; BNP, B-type natriuretic peptide; FDP, fibrin/fibrinogen degradation products.* | |

| **Supplementary Table S2. Sensitivity Analysis – Comparison of Complete-Case Analysis vs. Multiple Imputation for Key Predictors of 30-Day Mortality** | | |
| --- | --- | --- |
| **Predictor** | **Complete-Case OR (95% CI)** | **Multiple Imputation OR (95% CI)** |
| Age | 1.16 (1.12–1.21) | 1.15 (1.11–1.20) |
| cTnI elevated | 3.76 (1.87–7.56) | 3.68 (1.82–7.42) |
| BNP elevated | 6.59 (2.76–15.76) | 6.51 (2.71–15.62) |
| D-dimer elevated | 2.70 (1.17–6.24) | 2.68 (1.15–6.18) |
| *Multiple imputation by chained equations (MICE) was performed with 20 imputed datasets to assess the robustness of findings to missing data. Odds ratios (OR) are adjusted for age, Stanford classification, symptom-onset-to-admission time, systolic blood pressure, creatinine, treatment strategy, and all biomarkers. The results from multiple imputation were virtually identical to the complete-case analysis, with overlapping confidence intervals and no substantive changes in the direction or magnitude of associations, confirming the stability of our findings under different missing data assumptions. Abbreviations: OR, odds ratio; CI, confidence interval; cTnI, cardiac troponin I; BNP, B-type natriuretic peptide.* | | |

| **Supplementary Table S3. Spearman Correlation Matrix of Candidate Biomarkers** | | | | | | |
| --- | --- | --- | --- | --- | --- | --- |
|  | **cTnI** | **CK-MB** | **BNP** | **NT-proBNP** | **D-dimer** | **FDP** |
| **cTnI** | 1.000 | 0.104* | 0.130** | 0.130** | 0.186** | 0.088* |
| **CK-MB** | 0.104* | 1.000 | 0.171** | 0.171** | 0.147** | 0.004 |
| **BNP** | 0.130** | 0.171** | 1.000 | 1.000** | 0.203** | 0.192** |
| **NT-proBNP** | 0.130** | 0.171** | 1.000** | 1.000 | 0.203** | 0.192** |
| **D-dimer** | 0.186** | 0.147** | 0.203** | 0.203** | 1.000 | 0.191** |
| **FDP** | 0.088* | 0.004 | 0.192** | 0.192** | 0.191** | 1.000 |

**Correlation is significant at the 0.05 level (2-tailed).
**Correlation is significant at the 0.01 level (2-tailed).*

*"Spearman's rank correlation coefficients (ρ) are presented for all pairwise comparisons among the candidate biomarkers. Weak to moderate correlations were observed among most biomarkers (ρ range: 0.004 to 0.203). Notably, correlations between cTnI and CK-MB (ρ = 0.104, P = 0.013) and between D-dimer and FDP (ρ = 0.191, P < 0.001) were weak, indicating that collinearity does not explain the loss of independent prognostic significance for CK-MB and FDP. As expected, BNP and NT-proBNP showed perfect correlation (ρ = 1.000, P < 0.001), confirming biological redundancy between these markers. All VIFs remained below 3.5, confirming no significant multicollinearity. Abbreviations: cTnI, cardiac troponin I; CK-MB, creatine kinase-MB; BNP, B-type natriuretic peptide; NT-proBNP, N-terminal pro-B-type natriuretic peptide; FDP, fibrin/fibrinogen degradation products."*
